# To Physicians and Druggists

**Published:** 1878-06-20

**Authors:** 

**Affiliations:** Cincinnati, O.


### TO PHYSICIANS AND DRUGGISTS.

*Fluid Extract of Berberis Aquifolium.*—We have the true Berberis Aquifolium root in large amount. It was gathered expressly for us under the direct supervision of one of the ablest botanists west of the Mississippi river. We are now supplying the trade with our reliable fluid extract of Berberis Aquifolium in quantities. Our wholesale agents are W. R. Perrick, St. Joseph, Mo.; W. J. & L. A. Smith, Hartford, Conn.; J. O. Bosworth & Co., Denver, Colorado, and Pemberton, Samuels & Reynolds, Atlanta, Ga.

Our medicines are furnished by all these parties at our lowest price. Physicians throughout the South will please bear in mind the well-known Atlanta drug house above named, and order the extract which bears our label, thus securing the best. Respectfully,

Merrell, Thorp & Loyd,
Cincinnati, O.

George Stinson & Co., Art Publishers, Portland, Me.—We are in receipt from George Stinson & Co., art publishers, a floral cross, which for beauty and delicacy of finish we have not seen excelled. Their beautiful engravings are finding their way into many households.

